# Use composite coating of chitosan‐chia seed gum enriched with microliposomes of Bay laurel essential oil to increase the shelf life of quail fillets

**DOI:** 10.1002/fsn3.2578

**Published:** 2021-10-03

**Authors:** Motahareh Eslamian Amiri, Mohammad Ahmady, Peiman Ariaii, Leila Golestan, Azade Ghorbani‐HasanSaraei

**Affiliations:** ^1^ Department of Food Science and Technology Ayatolla Amoli Branch Islamic Azad University Amol Iran

**Keywords:** Bay laurel, Clevenger, combined coating, nanoliposome, phenolic compounds, Quail

## Abstract

In this study, the effect of composite chitosan‐chia seed coating (CH‐CG) with Bay laurel (*Laurus nobilis*) essential oil (BE) in two forms including free and nanocapsulated on the shelf life of quail fillets during the 16‐day refrigeration (4 ± 1℃) period was investigated. For this purpose, first, BE was extracted by Clevenger apparatus. Then, nanoliposomes BE were produced, and the properties of BE and nanoliposomes BE were investigated. In order to investigate the shelf life of quail, 6 treatments were produced including 1: control (C), 2: CH‐CG, CH‐CG+BE at 800 ppm, 3: CH‐CG+BE at 1600 ppm, 4: CH‐CG+nano BE at 800 ppm, 5: CH‐CG+nano BE at 1600 ppm, and periodically chemical parameters (peroxide value, free fatty acid, total volatile basic nitrogen, texture firmness, and chewing ability) and microbial (total viable bacteria (TVC) and psychrotrophic bacteria), and the effect of different treatments on control in *Escherichia coli* and *Staphylococcus aureus* inoculated populations in quail was also investigated. The BE had high antioxidant and antimicrobial properties. The particle size and microencapsulation efficiency of BE nanoliposome were 98.3 nm and 75.95%, respectively. The results of chemical and microbial analysis showed that in general, the coating with essential oil slowed down the increasing trend of oxidation and microbial indices compared to the control treatment and nanocapsulation of essential oil has increased its antimicrobial and antioxidant properties (*p* < .05). At the end of storage period, in all tests, treatments of 3, 4, and 5 had the allowed microbial and chemical range and they also inhibited the growth of these bacteria (*p* < .05). Overall, considering the higher sensory score of treatment 4 and economic efficiency, it seems that this treatment can be used as a natural preservative in the meat industry.

## Graphical abstract

The purpose of this study was to investigate the antioxidant and antimicrobial effects of combined coating of chitosan‐enriched chia seed gum with free and microencapsulated essential oil of Bay laurel to increase the shelf life of quail fillets during storage. According to the results, it seems that the combined coating of chitosan‐chia seed gum with nano essential oil of Bay laurel can be used as an active, safe, customer‐friendly, and affordable packaging for poultry without adverse environmental impact.

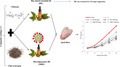

## 1 INTRODUCTION

In recent years, the meat industry has grown significantly due to the demand for meat products with very high nutritional properties as well as the development of meat products using new processing methods. All meat groups are a valuable source of protein and are considered the most complete food due to the presence of essential amino acids, minerals, and vitamins (Derakhshan et al., 2018). Among meat products, quail meat is the smallest halal poultry and is nutritionally more valuable than chicken due to its different vitamins. Low levels of cholesterol and fat in quail meat make vitamins valuable in this bird (Moawad et al., 2018).

At various stages of slaughter, transportation, storage, and processing, meat may be contaminated with a variety of microorganism. The most important factor that causes spoilage and shortens the life of meat is the growth of microorganism. Quail meat can be refrigerated for a limited time (4–5 days), as its muscle fibers are thin and prolonged storage can lead to spoilage, but its useful life can be extended by using other storage methods in combination with refrigeration (Aksu et al., 2014; Derakhshan et al., 2018; Moawad et al., 2018).

Therefore, the use of appropriate substances with antibacterial and antioxidant activity to improve the quality, increase the shelf life of meat, and at the same time prevent economic losses is necessary and useful (Farahmandfar & Tirgarian, 2020; Farahmandfar et al., 2018; 2020a). The use of antioxidants and microbial preservatives is one of the most important ways to prevent oxidative and bacterial spoilage of meat and meat products. In this regard, antioxidants and synthetic preservatives have been used for many years to control food spoilage. Today, consumers want to consume products of natural origin and with minimal processing. A safe and acceptable approach to increase food safety and shelf life is the use of plant essential oils (Sayyad & Farahmandfar, 2017; Sayyari et al., 2021, 2021,2021, 2021; Farahmandfar et al., 2019; Farahmandfar et al., 2020b; Moawad et al., 2018; Aksu et al., 2014). The Bay laurel with the scientific name of *Laurus nobilis* L. is an evergreen tree or shrub with a height of 15–20 m and two bases. In terms of chemical compounds, research has shown that alpha tocopherol is a major isomer in the vegetative organs of the Bay laurel plant, and the leaves contain flavonoids, Cescian terpenoid lactone, isoquinoline alkaloids, and phenolic acids. Also, the amount of alpha tocopherol in the leaves of Bay laurel plant leaves is very high and its roots contain a high amount of flavonoids (Tometri et al., 2020; Vilela et al., 2016). Recently, the improvement in the activity and effectiveness of nanoscale antimicrobial compounds has attracted more and more attention compared to the traditional mode (Bagheri et al., 2016). As Javadian et al. (2016) reported that microencapsulation of essential oils in liposomes improved their antimicrobial properties, liposomes are spherical particles composed of polar fats (such as phosphatidylcholine as well as phosphatidyl ethanolamine) or a mixture of polar fats with cholesterol or ergosterol. In addition, they are biodegradable, nontoxic, free from immunological hazards, and biocompatible compounds. They can microencapsulate antimicrobial and other functional compounds as a separate microenvironment and maintain their activity despite changes in the aqueous phase around them (Jimenez et al., 2014; Tometri et al., 2020).

Despite the high potential of plant essential oils, their use in maintaining the quality of food is limited mainly due to their aroma and severe toxic problems. To minimize the required doses, one of the interesting options is to use oral coatings and films as carriers of these natural compounds. Oral coatings have recently received more attention in the field of food preservation due to promising results (Valipour et al., 2017). Chitosan, after cellulose, is the most natural polysaccharide, which due to various properties such as biocompatibility, biodegradability, and nontoxicity, has many advantages and applications in various industries. However, pure chitosan coatings alone do not exhibit good mechanical properties, moisture barrier, and appropriate appearance properties. Also, the high price of chitosan compared to other biopolymers has led to finding a suitable solution for these coatings. Mixing chitosan with other polymers or gums can be a good way to improve its properties (Fasihi et al., 2017). Similarly, in this study, chitosan is combined with chia seed gum.

Chia seed (*Salvia hispanica* L) is a plant belonging to the mint family that contains 5% mucilage that can act as a soluble fiber. Today, chia seeds, as a rich source of nutrients and biological additives, are one of the favorite seeds in food industry technology and are cultivated and marketed in the Americas (Iglesias‐Puig and Haros, 2013). Chia seeds are also a rich source of synergistic and major antioxidants such as flavonols, chlorogenic acid, caffeic acid, myristin, quercetin, camphorl, as well as natural antioxidants such as tocopherols, phytosterols, and carotenoids. The presence of these compounds plays an important role in keeping the level of autoxidation low and increasing shelf life (Dick et al., 2015). According to the above, the purpose of this study was to investigate the antioxidant and antimicrobial effects of combined coating of chitosan‐enriched chia seed gum with free and microencapsulated essential oil of Bay laurel to increase the shelf life of quail fillets during storage.

## 2 MATERIALS AND METHODS

### 2.1 Raw material

The leaves of Bay laurel (*Laurus nobilis*) plant are prepared from the trees of Chabaksar city located in Gilan province in April 2020. Identification of the studied Bay laurel species was performed using the opinions of the professors of the Cultivation and Development Department of the Institute of Medicinal Plants. Excess parts of plants were separated and washed. Then, it was dried in a vacuum oven at 50℃ for 45 min and completely powdered by a shredder and kept at 25℃ until the experiment. Quail fillets were kept from the slaughterhouse in insulated containers near ice (0℃) and transported to the laboratory in polyethylene bags for filleting. The fillets were thoroughly rinsed with clean cold water until thoroughly cleaned of blood and other debris (Tometri et al., 2020). All the chemicals used were prepared by the German company Merck and had a degree of decomposition.

### 2.2 Preparation and analysis of Bay laurel essential oil

100 g of Bay laurel powder is mixed with one liter of distilled water, and essential oil extraction was performed for 3.5 h by Clevenger apparatus. The resulting essential oil was dehydrated using sodium sulfate and stored in dark containers at 4℃ until the experiment. Identification of compounds was performed using various parameters such as mass spectrum study and comparison of these spectra with standard compounds and information available in the computer library of GC/ MS (Shahbazi et al, 2016). The relative percentage of each of the constituents of the essential oil due to the area under its curve in the GC chromatogram was obtained by normalizing the surface and ignoring the response coefficients.

### 2.3 Measurement of total phenolic and flavonoid compounds

2.5 ml of 0.2 N Folin–Ciocalteu was added to 0.5 ml of each essential oil, and after 5 min, 2 ml of 75 g/L sodium carbonate solution was added. The adsorption of the mixture was read 2 h later at 760 nm by a spectrophotometer in front of Blank. Gallic acid is used as a standard for drawing calibration curves. Results were expressed in mg gallic acid per gram of essential oil (Ordonez et al, 2006).

Total flavonoid content was measured by aluminum chloride colorimetric method. 0.5 ml of essential oil with 1.5 ml of ethanol 95%, 0.1 ml of aluminum chloride 10%, 0.1 ml of 1 M potassium acetate, and 2.8 ml of distilled water were mixed together. After keeping the samples at room temperature for 30 min, the adsorption of the mixture was read at 415 nm. Quercetin was used to draw the standard curve, and the results were expressed in milligrams of quercetin per gram of essential oil. Thus, the base solutions of this substance with a concentration of 100 micrograms milliliters were prepared (Dewanto et al, 2002).

### 2.4 Measurement of antioxidant activity

#### 2.4.1 DPPH free radical scavenging test

2,2‐Diphenyl‐1‐picrylhydrazyl (DPPH) is a lipophilic radical having the maximum absorption at a wavelength of 517 nm. This test was determined based on the percentage of DPPH free radical scavenging by adding antioxidant compounds. This compound changes color from purple to yellow by taking electrons from antioxidants. DPPH absorption at 517 nm indicates the residual value (Maleki et al., 2016).

#### 2.4.2 Evaluation of ferric reducing antioxidant power (FRAP)

To measure ferric reducing antioxidant power (FRAP), 0.1 g of essential oil was homogenized with 5 ml of distilled water in a cold porcelain mortar in an ice bath. The resulting homogenate was filtered using Whatman No. 1 filter paper. Then, 1.5 ml of FRAP reagent (300 mM sodium acetate buffer with pH 3.6, ferric‐tripyridyl‐s‐triazine and ferric chloride) was added to 50 μl of the obtained essential oil. The resulting mixture was incubated for 30 min at 30℃. The absorption of the solutions at 593 nm compared to the control (including 50 μl of distilled water with 1.5 ml of FRAP reagent) was read. Ammonium ferrous sulfate was used as a control for comparison (Bougatef et al., 2009).

#### 2.4.3 Evaluation of Bay laurel essential oil antimicrobial activity

Liquid dilution method was used according to NCCLS recommendation. *Staphylococcus aureus* (PTTC, 1435) and *Escherichia coli* (PTTC, 1330) with an approximate concentration of 10^8^ cfu/ml were added to each of the test tubes at a rate of 0.2 ml. In the next step, Bay laurel essential oil solutions were prepared using Tween 80 (Merck, Germany) and distilled water in such a way that by pouring 0.2 ml of each of the solutions into the experimental tube containing the liquid culture medium and tested bacteria. The test tubes were then incubated at 37℃ for the bacteria. After 24 h, the lowest concentration at which no turbidity was observed was considered as minimum inhibitory concentration (MIC). In fact, the turbidity of the environment inside the erlens indicates the growth of bacteria, and the first test tube in which no turbidity was observed and was completely clear was considered as the MIC. After determining the MIC to determine the minimum bactericidal concentration (MBC) in completely sterile conditions, the contents of the Erlenmeyer flask, which was still clear after 24 h of incubation and did not show turbidity, 0.1 ml in petri dishes containing a culture medium suitable for any bacteria, surface culture was given. After 24 h of incubation at the appropriate temperature, the growth and nongrowth of bacteria were examined. The first concentration at which no growth was observed was considered as MBC (Tometri et al., 2020).

#### 2.4.4 Preparation of nanocapsule essential oil

Nanoliposomes were produced according to the method of Jimenez et al. (2014) with little change or modification. First, 2 g of lecithin and 2 g of tween 80 were mixed in 38 g of distilled water and shaken for 5 h. In the next step, 4 g of Bay laurel essential oil was added to the aqueous dispersion of lecithin and the whole mixture was sonicated for 600 s (1 s on and 1 s off) at 40 kHz and 40% of the device power. The produced nanoliposomes were stored in sterile bottles in the dark until use. Particle size distribution was measured using a laser light refraction device (Zetasizer nano zs. Malvern Co., England) (Joye et al., 2015).

The microencapsulation efficiency of polyphenols was determined according to the method described by Robert et al. (2015). 200 mg of microencapsulated was added to 2 ml of ethanol and stirred for one minute and then subjected to ultrasound for 20 min in two stages with 100% intensity and 20 kHz frequency. After this step, centrifugation was performed at 3000 *g* for 2 min. Alcohol can dissolve extracts outside the capsule without degradation. The amount of phenolic compounds in the supernatant was determined by the Folin–Ciocalteu method, and the absorption at 740 nm was determined by spectrophotometer. The percentage of encapsulation efficiency was calculated from the following equation:

$$\text{Encapsulation Efficiency}\;\left(\%\right)=\frac{w_{1}‐w_{2}}{w_{2}}\times 100$$

In this equation, *w*_1_ is the amount of essential oil in the upper liquid of a certain amount of nanocapsules, and *w*_2_ is the amount of essential oil added to prepare the same amount of nanocapsules, which is expressed in milligrams of gallic acid per gram of plant.

### 2.5 Release of essential oil in vitro

In the simulated environment with the stomach in the presence of pepsin, enzyme was performed according to the method of Chen et al. (2015). One gram of nanoliposome loaded with essential oil was dissolved in a laboratory tube in 1.5 ml of 0.1 N hydrochloric acid and incubated at 37℃ for 10 min at an impulse rate of 100 rpm. Then, 0.2 ml of pepsin solution with a concentration of 1 mg/ml in 0.1 N hydrochloric acid was added to start hydrolysis. Digestion was performed for 30 min, and then, the digested sample was centrifuged at 18,000 *g* and a clear supernatant was obtained. The amount of essential oil released was reported as a percentage of the total microencapsulated essential oil obtained from the encapsulation efficiency.

### 2.6 Coating preparation

The method of preparing the combined coating (chitosan + chia seed gum) enriched with free and microencapsulated essential oil of Bay laurel is obtained by dissolving 2% by weight/volume of chitosan in 1% volume/volume acetic acid. To better dissolve the chitosan, the solution was stirred with a magnetic stirrer for 3 h at room temperature.

To prepare a 2% w/v solution of chitosan coating, first 20 g of chitosan powder was added to one liter of distilled water and stirring was performed at a speed of 12000 *g* and then heated for 30 min at 70℃. The solution of chia seed gum was prepared at the level of 1.5 w/w by dissolving the gum in distilled water and stirring vigorously at a speed of 12000 *g* with a magnetic stirrer for 24 h at room temperature. In the next step, 200 ml of chitosan solution was slowly added to the gum solution and stirring was continued for 4 h. After this period, initially 0.2% v/v amount of essential oil, twin 80 as emulsifier with plant essential oil of Bay laurel at two levels of 800 and 1600 ppm in two free forms and nano in v/v form of chitosan solution was mixed mechanically and after uniformity, it was added to the coating solutions and stirred for two minutes with a homogenizer at 9000 rpm until the essential oils were evenly distributed in the coating matrix (Ojagh et al., 2010).

### 2.7 Preparation of samples

In order to impregnate the sample with quail fillet bacteria, 1 × 10^4^ *Staphylococcus aureus* and *Escherichia coli* were inoculated (in separate treatments). The inoculated meat samples were then completely homogenized. Inoculated quail fillets (80–100 g) were immersed in combined coating of (chitosan + chia seed gum (1.5%)) as well as combined coating (chitosan +chia seed gum (1.5%) enriched with free essential oil of Bay laurel (800 and 1600 ppm) and combined coating (chitosan + chia gum (1.5%)) enriched with microencapsulated essential oil of Bay laurel (800 and 1600 ppm), then removed from solution and allow the check water to be done for 30 s, then immerse in 2% calcium chloride solution for 30 s to induce cross‐linking in the coating. After coating, the samples were refrigerated (4 ± 1°C) and sampled completely randomly (3 samples from each section) at 4‐day intervals (0, 4, 8, 12, and 16) to count *Staphylococcus aureus* and *Escherichia coli* bacteria. Also, another part of the quail fillet (without bacterial inoculation) is coated as in the previous part. After coating, the samples are refrigerated and sampled completely randomly every 4 days (zero, 4, 8, 12, and 16) and were sampled for chemical and microbial tests (Tometri et al., 2020).

### 2.8 Chemical analysis

Measurement of peroxide value (PV): PV was determined based on Ye al. (2009) method, which is based on oxidation of iron II to iron III by hydroperoxides and formation of iron III‐thiocyanate complex.

Free fatty acid (FFA) of the fillet quail was determined by the procedure explained by AOAC (2005). Results were expressed as percentage of oleic acid.

The total volatile basic nitrogen (TVB‐N) of the quail was determined by the microdiffusion method as described by Javadian et al. (2016). Results were stated as mg N/100 g of samples.

Texture profile analysis (TPA) of each test sample was performed with three replications at room temperature with a histometer. For each treatment, three samples with a cylinder (2.5 cm in diameter) were separated from the middle of each fillet and subjected to a two‐stage pressure test. Samples were compressed to 40% of their original height with cylindrical spheres with a circular cross section 5 cm in diameter and a velocity of 5 mm/s (Rodríguez‐Carpena et al., 2012). Parameters of texture characteristics including hardness and chewing ability were identified.

### 2.9 Microbial analysis

In order to perform microbial tests, 5 g of the quail fillet sample taken from the sterile part was mixed with 45 ml of physiological saline solution and then the required dilutions were prepared. 1 ml of each dilution was used for culture by pour plate method. Culture samples incubated at 37℃ for 48 h to detect total viable bacterial and incubated for 7 days at 7℃ due to detect of psychrotrophic bacteria. The results were expressed as log CFU/g (Javadian et al., 2016).

*Staphylococcus aureus*: Baird Parker agar was used to count of *Staphylococcus aureus*. The results were expressed as log CFU/g (ISIRI 2001).

*Escherichia coli*: The CHROM agar ECC medium was used for enumeration of *E. coli*. The results were expressed as log CFU/g (Buller, 2004).

### 2.10 Sensory evaluation

The sensory quality of nonbacterial treatments cooked in the oven at 180℃ for 45 min was performed with the help of 15 semitrained evaluators. Simultaneously, it was evaluated using the graphical ranking scale method (Ozgul & Ozgul, 2017). The basis for selecting evaluators was physical health, having natural teeth, no smoking, no allergies and strong reluctance to consume the food under study and the correct diagnosis of color, odor, taste, and general acceptance of quail fillets on the first day of storage. Fresh water was made available to the evaluators for drinking between each diagnostic step.

### 2.11 Statistical analysis

All tests were performed with three replications and the data were given mean ± *SD*. Data analysis method was performed using analysis of variance Two‐way ANOVA (SPSS software18.0). Check for the presence of or no significant difference between the values obtained using the Duncan test at the level of 0.05 and figures were drawn with Microsoft Excel software.

## 3 RESULTS AND DISCUSSION

### 3.1 Evaluation of total phenolic and flavonoid compounds

Phenolic compounds are a large group of natural plant materials including flavonoids, tannins, and anthocyanins, which are usually found in fruits, vegetables, leaves, nuts, seeds, roots, and other parts of the plant. Flavonoids and other phenolic compounds are widely distributed in plants, and the diverse biological activity of these compounds, including their antioxidant and antimicrobial, has been reported in many studies. The amount of phenolic compounds in the present study was 388.9 ± 57.85 mg gallic acid/g essential oil, and flavonoid compounds were 210.4 ± 18.98 mg quercetin/g essential oil. Khodja et al. (2020) reported the phenolic compounds content of Bay laurel extract extracted with different solvents was between 380 to 510 mg gallic acid/g extract. Kivarak et al. (2017) stated that the phenolic compounds in the extract of Bay laurel extracted with different solvents were between 110 and 540 mg/g gallic acid and the amounts of flavonoid compounds were between 10 and 80 mg/g. As can be seen, there are differences in the amount of essential oil compounds in various studies. In general, the amounts of phenolic compounds can change according to the geographical area of growth, plant cultivar, plant age when preparing essential oil or extract, environmental and seasonal conditions, type of culture, harvest time, and finally plant genetic differences (Burt, 2004).

### 3.2 Investigation of antioxidant activity

**FIGURE 1:**
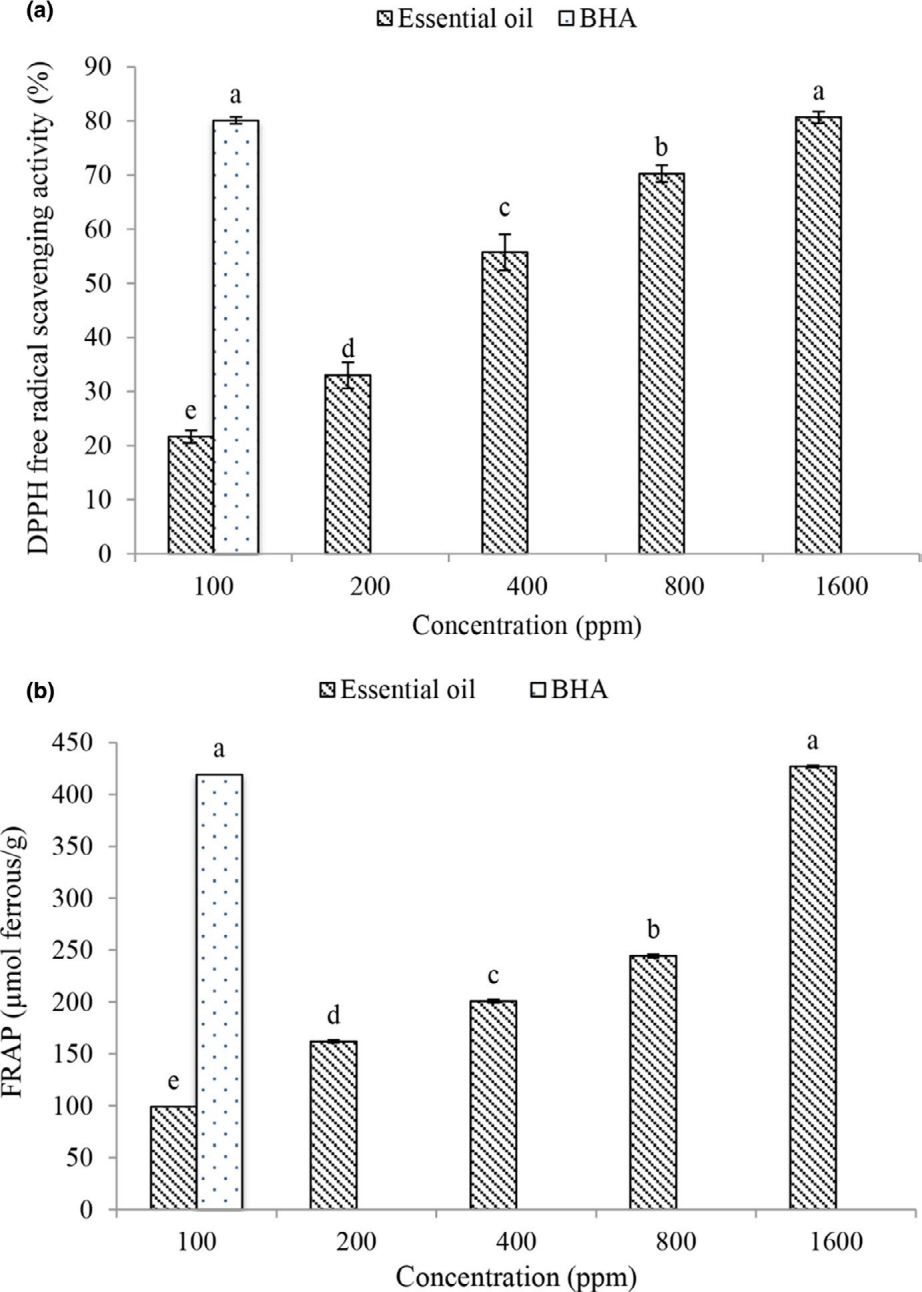
The amount of DPPH radical‐scavenging activity (a) and FRAP (b) of Bay laurel essential oil

The results of the present study showed that DPPH (Figure 1a) levels increased with increasing concentration of free radical activity. The essential oil at the concentration of 1600 ppm had the highest antioxidant activity and the amount of free radical activity of DPPH. Plant essential oils have antioxidant activity and high capacity for donating hydrogen atoms or electrons and free electrons due to their phenolic compounds. With increasing the concentration of phenolic compounds or the degree of hydroxylation of phenolic compounds, the radical inhibitory activity of the essential oil increases (Tometri et al., 2020; Rashidaie Abandansarie et al., 2019).

According to the results, the amount of ferric reducing antioxidant power (FRAP) (Figure 1b) was affected by the concentration of essential oil, and with increasing concentration, the amount of ferric reducing activity increased. The highest amount of ferric reducing power (FRAP) was observed at a concentration of 1600 ppm (89.17%). The antioxidant activity of reducing agents in essential oils is based on breaking the chain reactions of forming free radicals by donating electrons or hydrogen atoms (Bahrami Feridoni & Khademi Shurmasti, 2020).

In general, in both antioxidant tests, Bay laurel essential oil had antioxidant properties, and with increasing the concentration of essential oil, antioxidant activity increased, which is due to the higher phenolic compounds in this method. Phenolic compounds inhibit lipid oxidation reactions by donating electrons to free radicals. Flavonoids in the diet contain a group of catechols (2, 1‐dihydroxybenzene) through various mechanisms, including (1) acting as a free radical scavenger by giving a hydrogen atom or an electron, (2) binding to proteins and enzymes involved in the production of reactive oxygen species, (3) formation of complexes with transport metal ions capable of catalyzing the production of reactive oxygen species through redox cycles, and (4) reproduction of potent external antioxidants such as alpha tocopherols which are able to inhibit the oxidation of biological molecules (Khalili and Ebrahimzadeh 2015).

### 3.3 Investigating the Minimum Inhibitory Concentration (MIC) and Minimum Bactericidal Concentration (MBC)

The results of the present study showed (Table 1) that the Bay laurel essential oil has antimicrobial activity against both bacteria. Phenolic compounds in essential oils have antimicrobial properties. One of the reasons for the antimicrobial effects of essential oil is related to cell membrane changes due to the penetration of citral, citronellal, and geranyl acetate compounds and electrical imbalance of cell membranes and leakage of intracellular compounds out of the cell and eventually cell death. The Bay laurel plant is also due to its 1,8‐Cineole compound, which has antimicrobial properties. The antimicrobial properties of 1,8‐Cineole phenolic compound have been reported by other researchers (Caputo et al., 2017; Fidan et al., 2019).

**TABLE 1:** The amount of MIC and MBC of Bay laurel essential oil

Treatment	MIC (ppm)	MBC (ppm)
*Staphylococcus aureus*	358.66 ± 14.43^b^	458.33 ± 28.86^b^
*Escherichia coli*	441.66 ± 14.43^a^	666.66 ± 14.43^a^

Note

Different small letters in the same row represent significant difference (*p* < .05).

According to the results, Gram‐positive bacteria of *Staphylococcus aureus* had lower resistance. Resistance of gram‐negative bacteria to antibacterial agents with a hydrophilic surface of the outer membrane of bacteria is rich in lipopolysaccharide molecules and provides a barrier against the penetration of various antibiotic molecules, and it is also associated with periplasmic space enzymes that are able to break down imported molecules. Gram‐positive bacteria do not have such an outer membrane in the cell wall structure (Bozin et al., 2007; Bahrami Feridoni & Khademi Shurmasti, 2020).

### 3.4 Determination of constituents of Bay laurel essential oil

According to the results (Table 2), a total of 15 compounds with a total of 99.53% were identified. Most essential oil compounds included 1,8‐Cineole (56.45), Sabinene (13.55), α‐terpinyl acetate (9.35), and α‐Pinene (5.75). Bendjersi et al. (2016) also stated that the main compounds for the essential oils of Bay laurel include 1,8‐cineole (30.90%), sabinene (9.6%), α‐terpinyl acetate (7.8%), and linalool (9.9%–4.5%). In the study, the main constituents of the Bay laurel essential oil were 1,8‐Cineole. Minor differences in the amounts of compounds in various studies depend on the geographical area of growth, harvest time, and essential oil extraction methods from different organs and finally the genetic difference of the plant (Fidan et al., 2019; Mahdavi et al., 2018).

**TABLE 2:** Constituents of the Bay laurel essential oil

Row	Components	%
1	1,8‐Cineole	56.45
2	Sabinene	13.55
3	α‐terpinyl acetate	9.35
4	α‐Pinene	5.75
5	eugenol	4.33
6	p‐cymene	2.95
7	myrcene	2.22
8	β‐Pinene	1.85
9	eugenol eugenol	1.33
10	β‐Myrcene	0.78
11	γ‐terpinene	0.33
12	α‐terpineol	0.25
13	carvacrol	0.15
14	E‐caryophyllene	0.12
15	α‐ylangene	0
Total		99.53

### 3.5 Investigation of nanoliposome essential oil tests

The particle size of nanoliposome essential oil in the present study was 98.3 ± 2.56 nm. According to the results, the nanoliposome essential oils were small in particle size, which is due to the ability of the essential oils to create greater cohesion and compression between the nonpolar chains in the membrane vesicles (Valenti et al., 2001). The microencapsulation efficiency was equal to 75.95 ± 3.58%. In the structure of the liposome, there are two parts, hydrophilic and hydrophobic. Hydrophilic compounds are encapsulated in aqueous medium within liposomes, and hydrophobic compounds are enclosed between two phospholipid layers. Therefore, the phospholipid bilayer acts as a reservoir for the extract. Similar results about microencapsulation efficiency were observed by Tometri et al. (2020) and Sebaly et al. (2016) in terms of nanoliposomes. The results related to the release of essential oil from nanoliposomes showed that the release rate of essential oil increased significantly over time. During storage, liposomes gradually break down over time, leading to the release of substances from the liposome. These results with the results of Ghorani et al. (2017) while examining the effect of different biopolymers on microencapsulated saffron capsules showed that the release of effective compounds occurs over time and this trend is increasing during the storage period. These results are consistent with the results of their research.

### 3.6 Peroxide Value changes during storage

**FIGURE 2:**
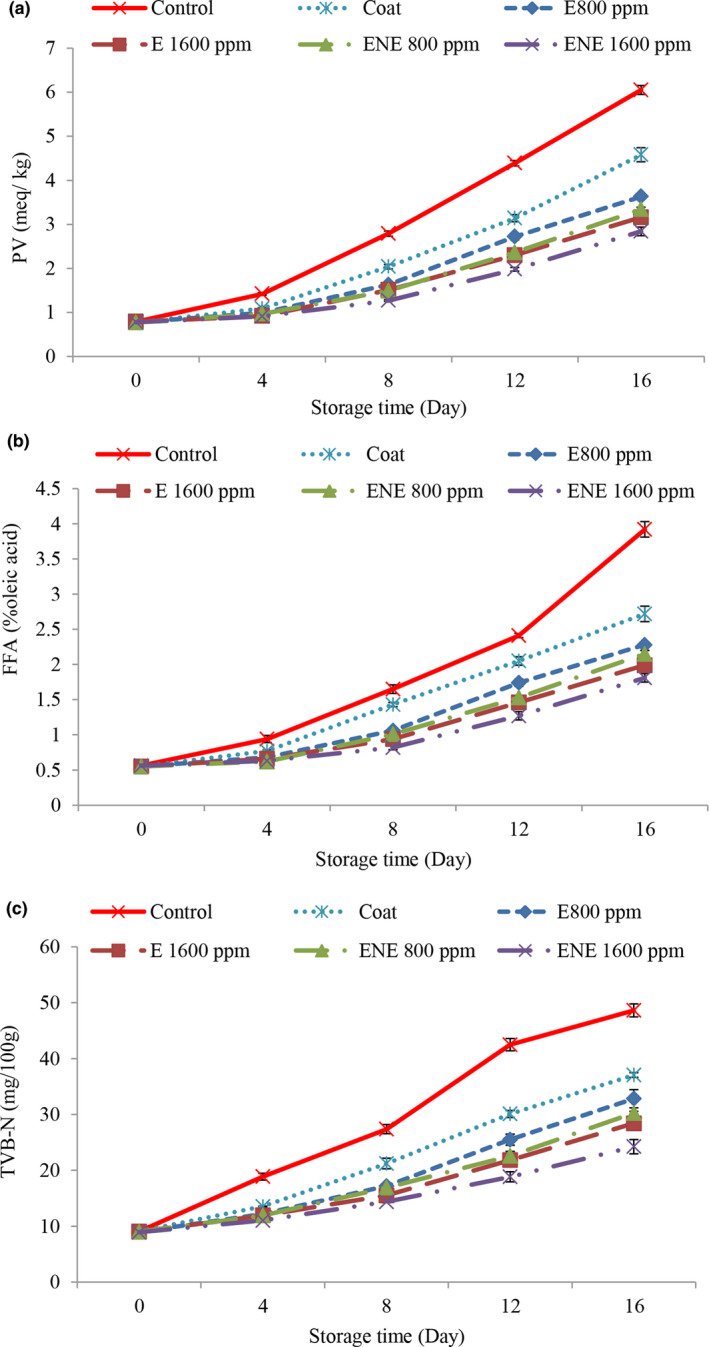
Changes in peroxide value (a), free fatty acid (b), and total volatile base nitrogen (c) of quail fillets during refrigerated storage

The results related to the peroxide value (PV) (Figure 2b) showed that it increased over time in all treatments and the comparison of the PV of the control sample with the rest of the treatments in different storage periods showed that the treatments containing the preservative increased the PV compared to the control treatment which slowed down it. The increasing trend slowed down the increasing trend of PV in treatments containing chitosan‐chia seed gum coating was slower than the control treatment. Bingöl et al. (2015) stated that chitosan has antioxidant power to retain fats in food. Valipour et al. (2017) also reported that coating phytophage fillets with chitosan slows the upward trend of PV compared to control treatment. Chia seeds are also a rich source of synergistic and major antioxidants such as flavonols, chlorogenic acid, caffeic acid, myristin, quercetin, Kaimfrol, as well as natural antioxidants such as tocopherols, phytosterols, and carotenoids. The presence of these compounds plays an important role in keeping the level of autoxidation low and increasing the shelf life of food (Dick et al., 2015; Shahhoseini et al., 2021). Also, the PV was lower in the treatments containing essential oil. The lower peroxide value was due to the phenolic compounds in the essential oil, because phenolic compounds prevent oxidation by inactivating fat free radicals and peroxy radicals. Some species of medicinal plants have different compounds but mainly contain polyphenols, which have antioxidant properties and therefore can increase the quality of quail (Mahdavi et al., 2018). By increasing the percentage of essential oil, this property increased. Similar results were reported by others (Jalali et al., 2016; Mahdavi et al., 2018).

The PV of nano‐essential oil treatment was lower than the other treatments. Encapsulation increases antioxidant activity, nanocapsulation protects the used hydrocolloids from environmental factors such as pH, oxygen, and light. Also, volatile molecules remain stable with this method and protect them from oxidative, optical, and volatile changes. Therefore, nanocapsulation has more potential to increase bioavailability, improve emission control, and accurately target biological compounds as a result of improving antioxidant activity (Bagheri et al., 2016).

The permissible amount of peroxide in meat for human consumption is 5 meq/kg (Yanar, 2007). At the end of storage period, the amount of PV in the control treatment was higher than the acceptable limit and in other treatments had a virtual range.

### 3.7 Check the amount of free fatty acid during the storage process

The highest amounts of free fatty acid (FFA) (Figure 2b) were observed in the control treatment on most days. Addition of coating + essential oil slowed down the increasing process of FFA, and by increasing the concentration of essential oil, better results were observed, which could be due to the antioxidant and antimicrobial effect of chitosan‐chia gum coating containing essential oil due to increased compounds such as −1 to 8. Cineol, which is present in the essential oil, reduced the process of hydrolysis of fatty acids and oxidation of the samples. Some researchers believe that the compounds in essential oils alter the structure of bacterial cell membranes, causing the secretion of various enzymes and nutrients, reducing microbial growth, and reducing fat hydrolysis (Nowrozi et al, 2013) and their synergistic effect with chitosan‐chia seed gum delays the process of hydrolysis and ultimately fat oxidation, which can reduce the oxidation indices in this treatment. Chitosan inhibits microbial growth by lowering the pH (pH 4.58–4.63) in the surface of quail meat, and the essential oil of Bay laurel, with its phenolic compounds, has largely prevented the growth of microorganisms and ultimately fat hydrolysis. The use of chitosan‐chia seed coating containing Bay laurel essential oil had a synergistic effect in reducing fat hydrolysis and ultimately oxidation (Tooryan & Azizkhani., 2020). Also, the results were better in relation to the treatments containing nanoliposome essential oil, so that on the 16th day of storage, the lowest values of FFA were observed in the treatment of nanocapsule essential oil with a concentration of 1600 ppm and the highest values were observed in the control treatment. The existence of significant differences in the amounts of FFAs in the sample of nanocapsule essential oil compared to the samples treated with free essential oil indicates a greater effect of nano essential oils in reducing the spoilage process.

### 3.8 Investigation of total volatile base nitrogen

According to the results, the highest total volatile base nitrogen (TVB‐N) (Figure 2c) was observed in the control treatment on most days. As the presence of bacteria in meat leads to the autolysis and degradation of proteins, the breakdown of compounds such as trimethylamine oxides, peptides, amino acids, and higher levels of bacterial load observed in the control samples could be a justification for increasing the amount of TVB‐N in them (Esmaili et al, 2020). Oral coatings act as antimicrobials and affect the amount of TVB‐N. The addition of essential oil also slowed down the increasing process of TVB‐N and had a positive effect in this regard with increasing concentration. It can be due to the reduced bacterial population of these treatments ability of bacteria to separate amines from nonvolatile nitrogen compounds or both factors attributed to the effect of the essential oil on the bacteria in quail fillets. As the concentration of essential oil increased due to the increase of phenolic compounds, its antibacterial effect also increased (Frangos et al., 2010). The values of TVB‐N in the treatments containing nano‐essential oil were lower than the other treatments. The reason for this is to increase the antibacterial properties of essential oils after encapsulation or to maintain the stability of antibacterial properties for a longer period after encapsulation. Ramezani et al. (2019) stated that the permissible amount of TVB‐N in quail for human consumption is 30 mg/g. Accordingly, at the end of the storage period, treatments containing essential oils with a concentration of 1600 ppm and nano‐essential oils with concentrations of 800 and 1600 ppm had a permissible range.

### 3.9 Texture properties during storage

**FIGURE 3:**
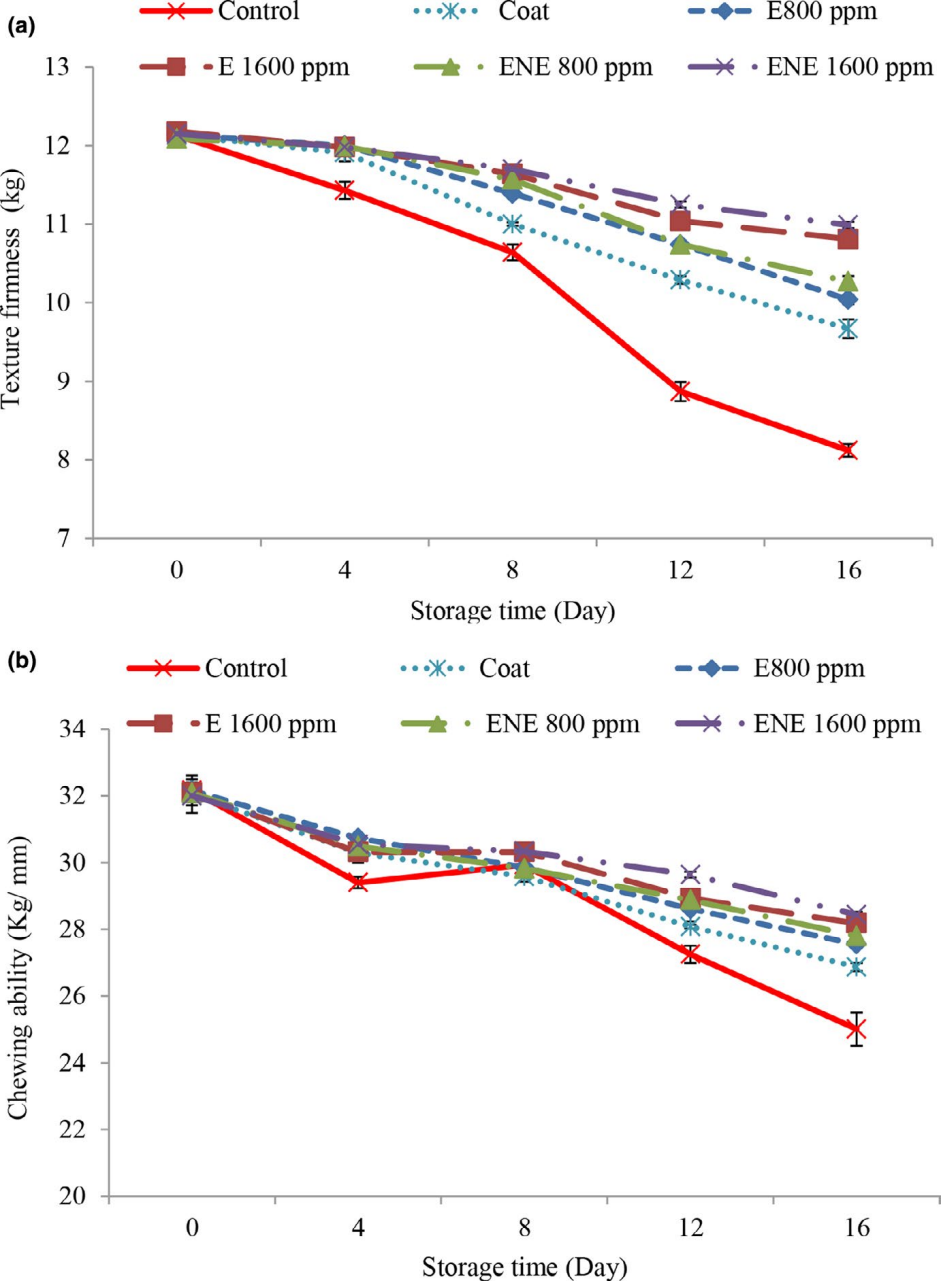
Changes in texture firmness (a) and chewing ability (b) of quail fillets during refrigerated storage

The results related to texture firmness (Figure 3a) and chewing ability (Figure 3b) were consistent, so that with increasing time, the values of texture firmness and chewing ability decreased in all treatments and these changes were more in the control treatment. Oxidation of fats and denaturation of proteins leads to changes in muscle integrity, denaturation, and accumulation of myofibrillar proteins in quail fillet meat. Addition of coating and essential oil slowed down the process of changes in meat texture, which is probably due to the protective effect of coating along with essential oil on lipid oxidation, and the treated samples had relatively higher hardness than the control sample (Masoumi et al., 2018). With increasing the concentration of essential oil, better results were observed and also the results were better in relation to treatments containing nanoliposome essential oil. This indicates an increase in the antioxidant properties of the essential oil after nanoliposme.

### 3.10 Total viable bacteria and psychrotrophic bacteria during storage

**FIGURE 4:**
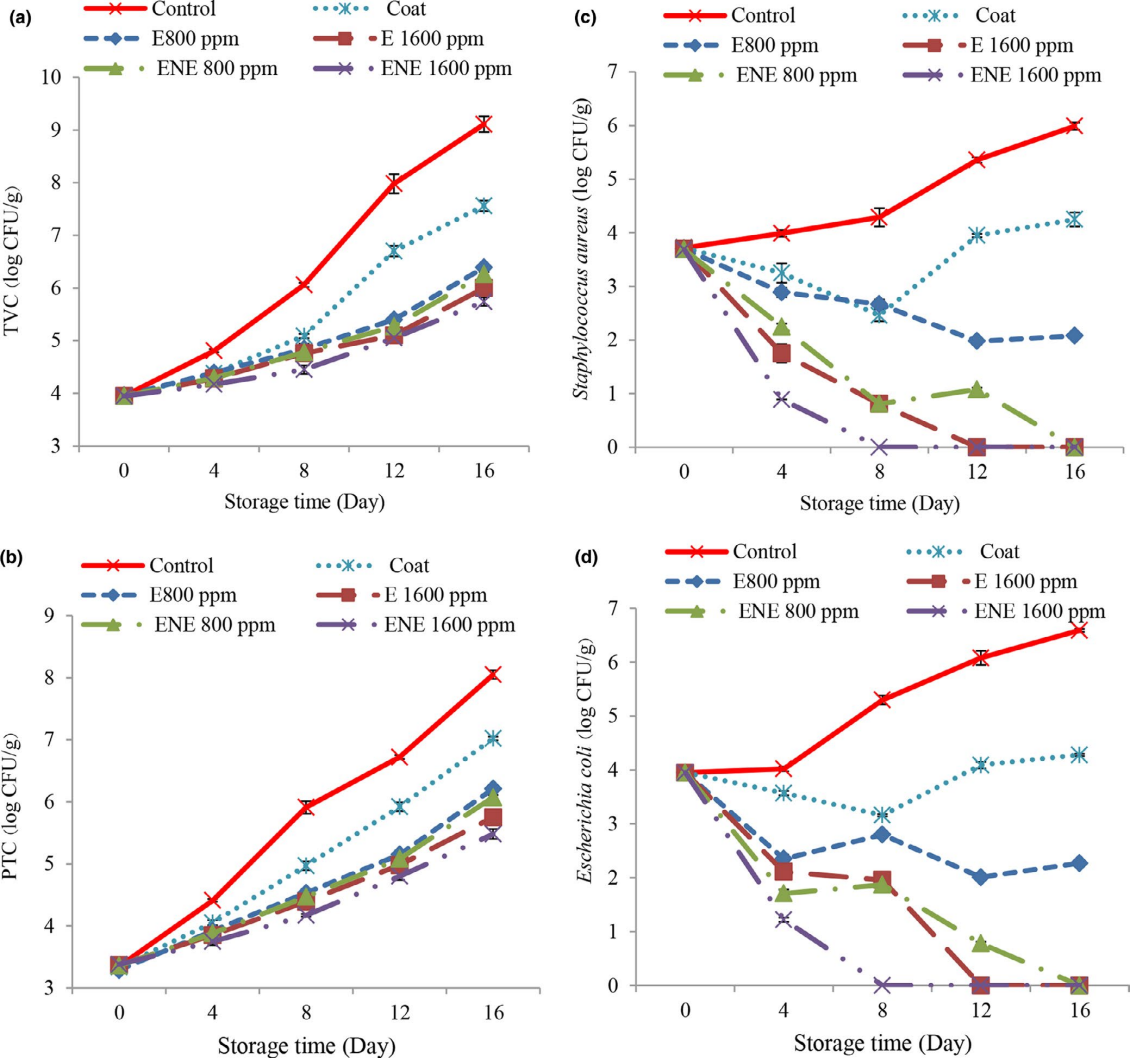
Microbiological counts of different bacteria in the quail fillets during refrigerated storage. Total viable count (a), psychrotrophic bacteria count (b), *Staphylococcus aureus* (c), and *Escherichia coli* (d)

In the present study, the results of total viable bacteria (TVC) (Figure 4a) and psychrotrophic bacteria (PTC) (Figure 4b) were consistent with each other, so that according to the results in most days, the highest amounts of PTC and TVC were observed in the control treatment. Coating with chitosan‐chia seed gum slowed the growth of bacteria. One theory attributes this effect of chitosan to the presence of positively charged amino groups that bind to large negatively charged molecules on the surface of the microbial cell, leading to rupture of the bacterial cell membrane, leakage of intracellular material, and eventually its death. (No et al., 2007). Chia seeds are also a source of antimicrobial properties due to the presence of compounds such as flavonols, chlorogenic acid, caffeic acid, myristin, quercetin, and camphor (Dick et al., 2015). The addition of essential oil also slowed down the process of increasing TVC and PTC. Lower these parameter in treatments containing essential oil can be due to phenolic compounds such as cineole. ATP withdrawal leads to depletion of cell energy storage and cell death (Jan Khan et al., 2017). The antimicrobial properties of essential oil depend on the concentration used, and as the concentration increases, their antimicrobial properties increase (Jalali et al., 2016; Rashidaie Abandansarie et al., 2019). Also, the results were less in relation to the treatments containing nano‐essential oil than other treatments. This is due to the increased antimicrobial properties of the coatings after nanocapsulation. A possible explanation for this could be that the use of nanocapsulation better preserves bioactive compounds, thus protecting food more effectively against oxidation and the growth of pathogenic and spoilage microorganisms (Gortzi et al., 2006). Increased antimicrobial properties of natural preservatives after nanocapsulation by various carriers have also been reported by other researchers (Javadian et al., 2016; Bagheri et al., 2016; Tometri et al., 2020). The allowable amount of TVC and PTC has been suggested 7 log CFU/g for poultry meat (ICMSF, 2005). At the end of the storage period, the amount of bacteria in all samples except the control treatment and cover treatment had an acceptable level.

### 3.11 Evaluation of changes in *Staphylococcus aureus* during storage

With increasing time, the levels of *Staphylococcus aureus* (Figure 4c) increased in the control treatment and decreased in most treatments. In fact, this indicates the antimicrobial properties of chitosan‐chia seed gum. It is believed that chitosan exerts its antimicrobial properties by destroying the cellular structure of spoilage and pathogenic microorganisms. In fact, chitosan, by altering the permeability of the cytoplasmic membrane, causes the release of electrolytes and intracellular protein components and ultimately leads to the death of microorganisms (Valipour et al., 2017). Also, the addition of essential oil increased the antimicrobial properties. The chemical structure of phenolic compounds affects their antimicrobial mechanism, and the hydroxyl groups in phenolic compounds have an important effect on the antimicrobial properties of essential oils and plant extracts. The presence of an active hydroxyphenolic group has made these compounds easily able to form hydrogen bonds with active sites of enzymes (Mahdavi et al., 2018). These compounds usually disrupt the cytoplasmic membrane, breaking and disrupting the proton kinetic force, electron current, and active conduction, causing coagulation of cell contents (Mahdavi et al., 2018) as well as results related to treatments containing microencapsulated essential oils was better, so that from the 8th day of storage, no *Staphylococcus aureus* bacteria were observed in the treatment of nanocapsulated essential oil with a concentration of 1600 ppm, which indicates an increase in the antimicrobial properties of the essential oil after nanoliposome.

### 3.12 Evaluation of changes in *Escherichia coli* during storage

With increasing time, the levels of *Escherichia coli* (Figure 4d) in the control treatment increased and in other treatments had a decreasing and increasing trend. According to the results of statistical analysis in most days, the highest values were observed in the control treatment. With increasing the concentration of essential oil, better results were observed and also the results were better in relation to the treatments containing microencapsulated essential oil, so that from the 8th day of storage, no *Escherichia coli* bacteria were observed in the treatment of nanocapsulated essential oil with 1600 ppm. The antimicrobial properties of Bay laurel plant against *Escherichia coli* have also been reported in the study of other researchers (Caputo et al., 2017; Fidan et al., 2019).

### 3.13 Investigation of sensory characteristics at the beginning of the maintenance period

**FIGURE 5:**
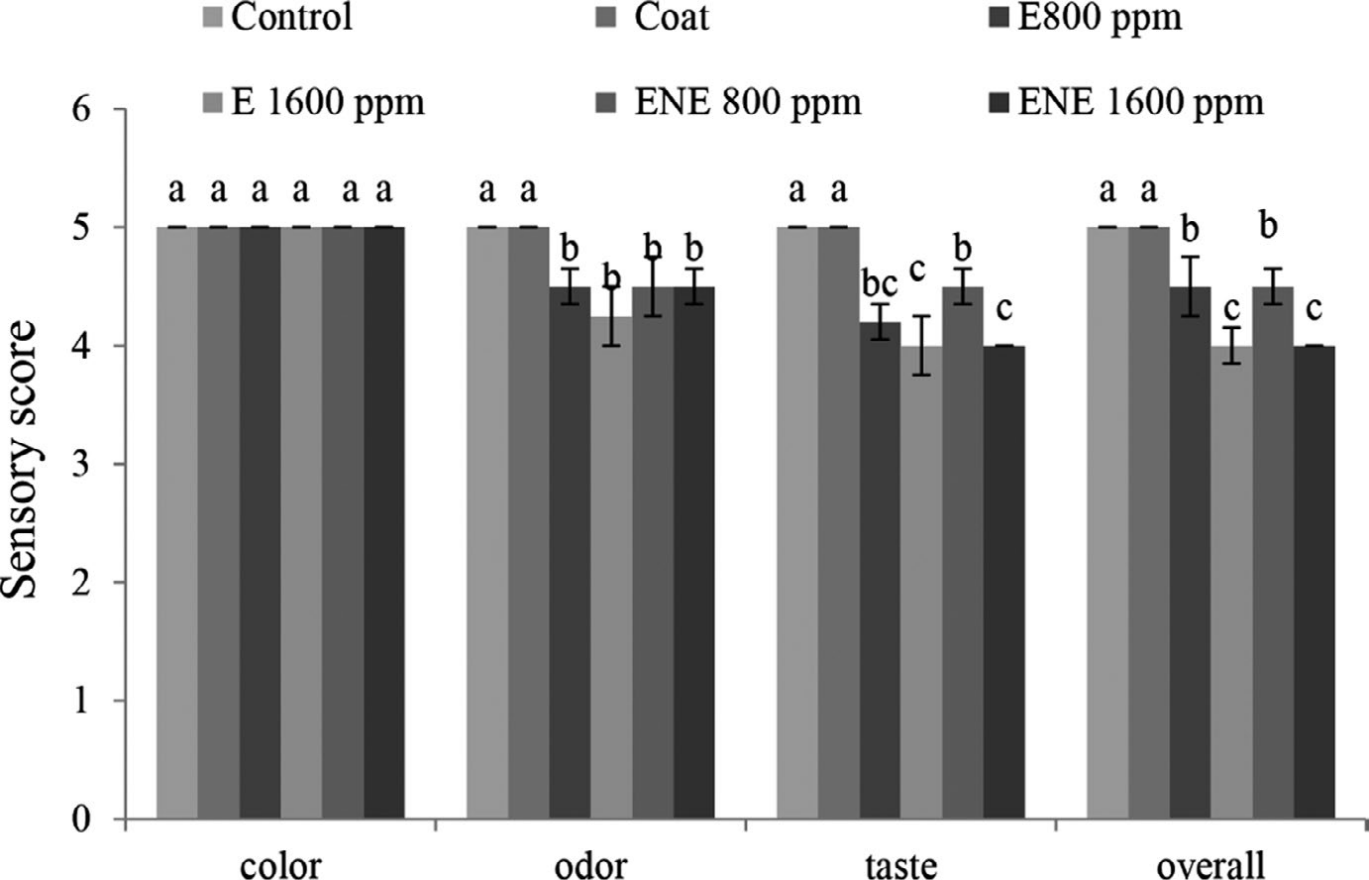
Sensory evaluation of quail fillets at the beginning of storage

According to the results (Figure 5a), with the addition of preservatives, sensory score was significantly reduced, but all treatments had sensory scores approved by the evaluators. Tometri et al. (2020) also reported that the use of microencapsulated extract and free Bay laurel extract reduced the sensory properties compared to the control treatment. In general, all treatments in their study had a sensory score approved by the evaluators.

## 4 CONCLUSION

Bay laurel essential oil has phenolic and flavonoid compounds and high antioxidant and antimicrobial properties. Also, the results related to the shelf life of quail showed that the combined coating of chitosan‐chia seed gum along with essential oil slowed down the increasing trend of oxidative and microbial spoilage indices over time, and better results were observed with nanoliposome of essential oil. In general, it seems that the composite coating of chitosan‐chia seed with nano bay laurel essential oil can be used in the packaging industry. Further studies with other poultry and other composite coatings with native nano essential oil may provide promising results.

## CONFLICT OF INTEREST

The authors declare that they do not have any conflict of interest.

## AUTHOR CONTRIBUTIONS

**Motahareh Eslamian:** Investigation (equal); Writing‐original draft (equal). **Mohammad Ahmady:** Supervision (equal). **PEIMAN ARIAII:** Software (equal). **Leila Golestan:** Resources (lead). **Azade Ghorbani‐HasanSaraei:** Writing‐review & editing (lead).

## STUDIES INVOLVING HUMAN AND ANIMAL SUBJECTS

Human and animal testing is unnecessary in this study.

## INFORMED CONSENT

Written informed consent was obtained from all participants.

## Data Availability

All the data used in this study can be made available upon reasonable request.
